# Comprehensive genomic analysis of early and late-onset hepatocellular carcinoma

**DOI:** 10.1016/j.gendis.2023.06.005

**Published:** 2023-07-12

**Authors:** Jialin Yang, Chao Chen, Song Wang, Yaru Zhang, Jiaohui Pang, Yajun Wang, Qiuxiang Ou, Hao Jiang, Juying Liu

**Affiliations:** a Department of Radiation Oncology, Sichuan Cancer Hospital & Institute, Sichuan Cancer Center, School of Medicine, University of Electronic Science and Technology of China, Chengdu, Sichuan 610041, China; b Jinling Hospital, Department of Oncology, Nanjing Medical University, Nanjing, Jiangsu 210002, China; c Geneseeq Research Institute, Nanjing Geneseeq Technology Inc., Nanjing, Jiangsu 210000, China; d Department of Oncology, Haian Hospital of Traditional Chinese Medicine, Haian, Jiangsu 226600, China; e Interventional Therapy Department, Jiangsu Cancer Hospital & Jiangsu Institute of Cancer Research & The Affiliated Cancer Hospital of Nanjing Medical University, Nanjing, Jiangsu 210009, China

Hepatocellular carcinoma (HCC) is the major histologic type of primary liver cancer, accounting for approximately 75% of liver cancer cases.^1^ Despite many advancements in its treatment, the prognosis and drug response of HCC patients are dismal. Therefore, there is an unmet clinical need to explore genomic aberrations underlying early- and late-onset HCC, which might facilitate drug discovery and provide personalized biomarker-driven treatment options for these patients. This study aimed to identify biomarkers associated with early-onset (EO) and late-onset (LO) HCC with comprehensive genomic profiling.

We retrospectively analyzed next-generation sequencing data of baseline tumor samples from 222 patients histologically diagnosed with HCC (Fig. S1). The median age of this cohort of patients was 55 years old (range: 19–80), and most patients had no family history of cancer (Table S1). A total of 47 patients (47/222, 21.2%) were hepatitis B virus (HBV)-positive while a significant proportion of patients were missing the information. Of note, the age cut-point used in this study that classified early-onset and late-onset HCC was 40 years for males and 50 years for females, in accordance with the 2018 American Association for the Study of Liver Diseases (AASLD) guideline for the management of HCC.^2^ The male incidence rate of LO patients was significantly higher than that of EO patients (88.6% *vs*. 56.8%, *P* < 0.001).

Comprehensive genomic profiling of baseline tumors revealed that the top frequently mutated genes were *TP53* (63.5%) and *telomerase reverse transcriptase* (*TERT*, 32.0%), followed by *MCL1* (21.1%), *MYC* (17.6%), and *ARID1A* (15.3%) (Fig. 1A). Telomerase reactivation in HCC is a key event of malignant transformation that stimulates the uncontrolled proliferation of tumor cells.^3^ Notably, *TERT* promoter mutations accounted for the most common somatic alteration observed among all patients with aberrant *TERT* activation (∼85.5%), whereas *TERT* amplifications were identified in 5.4% of all cases. The cell cycle control pathway emerged as the major dysregulated pathway in HCC, including genetic variants of *TP53* (70.7%), *CDKN2A* (13.5%), *CTNNB1* (13.1%), *RB1* (11.3%), and *CDKN2B* (8.1%). Prior studies have reported higher frequencies of *TERT* activation mutations (∼70%) and *CTNNB1* mutations (∼30%), as well as a lower frequency of *TP53* alterations (∼30%) compared with our study.^4^ However, next-generation sequencing methodologies (targeted gene panel in our study *vs*. WES/WGS in others) and ethnic differences of patients may lead to disparities in variant detection among studies. Besides, consistent with previous findings, our study also revealed aberrations in other key signaling pathways, including the phosphoinositide 3-kinase (PI3K) signaling [*PTK2* (13.5%), *TSC2* (10.4%), *AKT3* (8.6%)], chromatin remodeling [*ARID1A* (15.3%), *ARID2* (7.2%)], MAP kinase signaling [*NTRK1* (13.1%), *KRAS* (6.3%)], receptor tyrosine kinase (RTK) signaling (*DDR2*, 14%), and Wnt signaling (*APC*, 6.3%).

**Figure 1:**
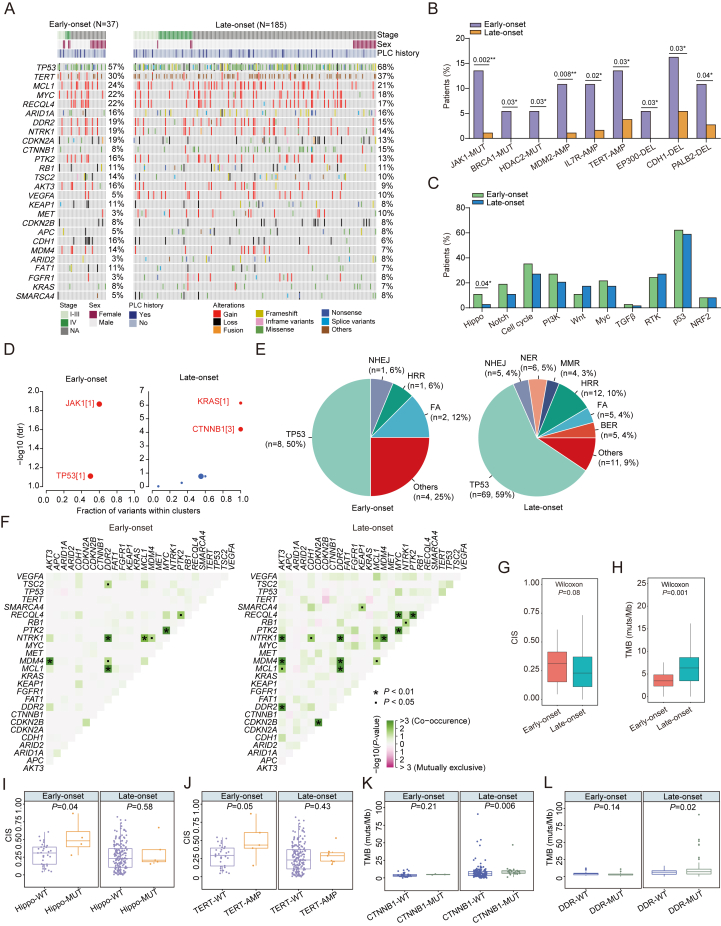
Genomic alteration associated with early- and late-onset hepatocellular carcinoma (HCC). **(A)** Co-mutation plot of early- and late-onset HCC patients. Each column represents one patient. The clinical characteristics of patients are shown at the top, and mutation frequencies of each gene in the patient subgroup are listed to the right of the plot. Only genetic alterations with a mutational frequency ≥5% are shown. **(B)** The bar plots illustrating the proportion of early- and late-onset HCC patients harboring genetic alterations with a mutational count ≥2 reads. **(C)** The bar plots illustrating the proportion of HCC patients in the two subgroups harboring genetic alterations in the relevant pathways. **(D)** The plot demonstrating driver genes significantly enriched in early- and late-onset HCC patients. **(E)** Distribution of *TP53* and DNA damage repair pathway alterations in subgroup patients. **(F)** Somatic interaction analysis of the top frequently mutated genes in early- and late-onset HCC patients. Statistically significant pairs are indicated with dots and asterisks if the FDR-adjusted *P*-value is < 0.05 or <0.01, respectively. **(G, H)** The box plots illustrating the chromosomal instability score (CIS) (G) and the tumor mutation burden (TMB) (H) in subgroup patients. **(I, J)** Distribution of CIS in patients harboring Hippo pathway genetic alterations (I) or *TERT* amplification (J) compared with corresponding wild-type patients within two patient subgroups. **(K, L)** Distribution of TMB in patients positive for *CTNNB1* mutations (K) or DDR pathway genetic alterations (L) compared with corresponding wild-type patients within two patient subgroups. NHEJ, non-homologous end joining; MMR, mismatch repair; HRR, homologous recombination repair; FA, Fanconi anemia; BER, base excision repair.

At the individual gene level, we identified several genetic alterations more prevalent in EO but not LO patients with HCC, including *JAK1*, *BRCA1*, and *HDAC2* mutations, amplifications of *MDM2*, *IL7R*, and *TERT*, as well as deletions of *EP300*, *CDH1*, and *PALB2* (Fig. 1B). Pathway-level enrichment analysis showed that Hippo pathway-related alterations were more frequently found in EO compared with LO patients (10.8% *vs*. 2.7%, *P* = 0.04; Fig. 1C). Although variants in the Wnt and RTK signaling pathways were more abundant in HCC patients with a late onset of the disease, the difference was not statistically significant. In addition, we found that known cancer drivers, specifically *Janus kinase 1* (*JAK1*) and *TP53*, were primarily identified in EO patients, while *KRAS* and *CTNNB1* were predominantly found in LO patients (Fig. 1D). As the DNA damage repair (DDR) pathway modulates cancer risk, progression, and therapeutic response across various cancer types by maintaining human genome stability, we then assessed the distribution of different DDR functional pathways in HCC. Although *TP53* was identified as the most frequently mutated gene in both patient subgroups, the distribution patterns of other DDR pathway genes significantly differed between EO and LO patients. As shown in Figure 1E, genetic alterations in the nucleotide excision repair, mismatch repair, and base excision repair pathways were only observed in LO patients but not EO patients. However, the distribution difference was not statistically significant due to the limited number of mutations being examined.

We also analyzed somatic mutation interactions to investigate genetic alterations that exhibit co-occurrence or mutual exclusivity. We found evidence for co-occurring mutations in 9 and 17 gene pairs of early- and late-onset HCC patients, respectively (Fig. 1F). Of these, 66.7% (6/9) and 47.1% (8/17) of interactions were shared between the two patient subgroups. On the other hand, *TP53* mutations rarely co-exist with *ARIDIA* mutations in early-onset patients. In contrast, *TP*53 mutations were likely mutually exclusive to *ARIDIA* mutations and *CDKN2B* deletions in late-onset patients with HCC. Moreover, various mutual exclusive gene interaction pairs were identified in late-onset patients, including *TERT* ∼ *CDKN2B*, *RB1*∼*CDKN2A*, and *CTNNB1*∼*APC*, although none of them exhibited statistical significance.

Chromosome instability, a source of genetic variation in either altered chromosome number or structure, has become an active area of investigation for its implications in determining genetic heterogeneity in tumors and possibly therapeutic responses to reveal treatment efficiency.^5^ Across the study cohort, we observed a slightly higher chromosomal instability score (CIS) in EO patients than in LO patients (median: 0.31 *vs*. 0.23, *P* = 0.08; Fig. 1G). Interestingly, tumor mutation burden (TMB) showed an opposite trend, with higher TMB identified in LO than in EO patients (median: 6.5 *vs*. 3.5 muts/Mb, *P* = 0.001; Fig. 1H). Consistently, the percentage of TMB-H patients was significantly higher in LO patients than in EO patients (27.6% *vs*. 10.8%, *P* = 0.04; Fig. S2A). In addition, microsatellite instability patients were exclusively identified in LO patients (2.7% *vs*. 0%, *P* = 0.59; Fig. S2B). As TMB serves as a surrogate measure of neo-antigenicity, which is associated with the stimulation of antitumor immunity, our findings suggest that patients with late-onset HCC are likely to benefit from immunotherapy due to a higher tumor mutational burden.

Next, we assessed whether the genetic difference could affect cancer biology and response to therapy. As shown in Figure 1I, we noticed that patients bearing Hippo pathway alterations showed significantly higher CIS compared with wild-type patients in the EO subgroup (median: 0.49 *vs*. 0.29, *P* = 0.04), while no significant difference was found in the CIS of LO patients with or without these alterations (median: 0.17 *vs*. 0.24, *P* = 0.58). A similar CIS distribution was observed in *TERT*-amplified EO and LO patients (Fig. 1J), with a higher CIS observed in *TERT-*amplified EO patients compared with *TERT*-wildtype EO patients (median: 0.44 *vs*. 0.29, *P* = 0.05). In contrast, no significant difference in CIS between *TERT*-amplified and *TERT*-wildtype LO patients was observed (median: 0.29 *vs*. 0.23, *P* = 0.43). No significant correlation between other specified alterations and CIS was identified within EO and LO subgroups (Fig. S3).

Similarly, we performed TMB analyses in subgroup patients. As a result, *CTNNB1*-mutated patients were associated with higher TMB in the LO subgroup (median: 8.4 *vs*. 5.8 muts/Mb, *P* = 0.006; Fig. 1K) but not in the EO subgroup (median: 4.8 *vs*. 3.2 muts/Mb, *P* = 0.21). Additionally, higher TMB was associated with DDR-altered LO patients compared with DDR-wildtype LO patients (median: 10.4 *vs*. 5.9 muts/Mb, *P* = 0.02; Fig. 1L). None of the other genetic alterations or oncogenic pathways significantly contributed to TMB differences within the two patient subgroups (Fig. S4).

Due to the lack of survival data, we explored the clinical association between the genomic features and the patient's overall survival (OS) using The Cancer Genome Atlas (TCGA) dataset consisting of 159 Asian patients with HCC (Fig. S1 and Table S2). A similar clinical context was observed between the TCGA dataset and this cohort of patients (Table S3). No significant difference was observed in the traits including sex (*P* = 0.29) and disease onset (*P* = 0.39) between the two cohorts. It is worth noting that 75.2% (167/222) and 78.8% (175/222) of patients in the current study cohort were missing the clinical stage and risk factor information, respectively. Besides, the family history of cancer differed significantly between the two cohorts (*P* < 0.001), but it had no significant impact on the OS of patients in the univariate survival analysis (hazard ratio: 0.58, 95% confidence interval: 0.21–1.65, *P* = 0.31) (Table S4). In the TCGA dataset, LO-HCC patients had a significantly higher TMB than EO-HCC patients (median: 2.8 *vs*. 1.6 muts/Mb, *P* < 0.001; Fig. S5A), which was consistent with that in our study cohort. Several genetic biomarkers were found negatively associated with patient's OS in HCC, including high TMB (*P* < 0.001), *EP300* deletion (*P* = 0.003), *TP53* deletion (*P* = 0.02), *IL7R* amplification (*p* = 0.04), and Hippo pathway variants (*P* = 0.02; Table S4). Among these, only TMB remained as an independent biomarker significantly associated with an unfavorable prognosis in HCC patients (hazard ratio: 2.65, 95% confidence interval: 1.37–5.1, *P* = 0.004) (Fig. S5B). The age at diagnosis had no significant impact on patient's survival (hazard ratio: 1.6, 95% confidence interval: 0.58–4.5, *P* = 0.35; Table S4), despite a trend of shorter OS in late-onset HCC patients versus early-onset patients was observed (Fig. S5C). We reasoned that the limited sample size of early-onset HCC patients might impair the statistical power of our tests. On the other hand, TMB-H patients had a significantly shorter OS than TMB-L patients (median: NA *vs*. 53.3 months, *P* < 0.001; Fig. S5D). Additionally, the OS was significantly different between HCC patients with TMB-H and with TMB-L regardless of disease onset time (*P* = 0.004; Fig. S5E).

Lastly, we referred to the OncoKB database and previously reported clinical trials to identify potential biomarker candidates that might facilitate targeted therapy for HCC (Table S5). Here, we found a total of 32 actionable genes in 54.1% and 50.3% of EO and LO patients, respectively. Since we have previously found that *MDM2* amplifications were more likely enriched in early-onset HCC patients. Therefore, targeting MDM2 may have potential clinical implications as an actionable therapeutic target for subgroup patients.

Collectively, we showed distinct molecular characteristics of HCC patients with an early- or late-onset of the disease, which might be relevant to cancer biology and response to therapy. Our findings suggest that genome instability caused by aneuploidy or structural changes in chromosomes might be associated with HCC at earlier ages, whereas accumulation of genetic aberrations resulting in high tumor mutational load may contribute to the development of late-onset HCC. Our findings suggest that *CTNNB1*-mutated or DDR pathway-altered late-onset patients who had high TMB may benefit from immunotherapy and that *MDM2* amplification was significantly enriched in early-onset patients, indicating that *MDM2* inhibition might be a promising biomarker-driven precision treatment strategy for these patients. Moreover, implementing screening programs to identify genetic alterations that are more prevalent in early-onset HCC patients can aid in the detection of the disease, which may ultimately improve patient survival outcomes through timely intervention and curative treatments.

Despite these promising results, there are limitations to this study. First, we acknowledge that hepatitis B and C viruses are independent risk factors for cirrhosis development, which remains the most important risk factor for the development of HCC regardless of etiology. However, only 21.2% of patients in our study were HBV-positive while a significant proportion of patients lacks the information mainly due to the retrospective nature of the study and the fact that patients came to the participating tertiary cancer centers with incomplete clinical information. Enormous efforts should be made to standardize protocols for patient clinical information transfer. Second, our study is limited by the lack of survival data on patients. Therefore, it is imperative for future studies to address these issues and conduct more comprehensive assessments to better elucidate the genomic characteristics of patients with early- and late-onset HCC.

## Ethics declaration

The study was approved by the Medical Ethics Committee of Nanjing Geneseeq Medical Laboratory (NSJB-MEC-2023-02). Written consent was obtained from each patient. The informed consent form was obtained from each patient.

## Author contributions

JLY and CC designed the study. JLY, CC, SW, and YRZ performed data analysis. JLY, CC, SW and QXO prepared the manuscript. HJ and JYL supervised the research. All authors read and reviewed the final manuscript.

## Data availability

The datasets generated and/or analyzed during this current study are available from the corresponding author upon reasonable request.

## Conflict of interests

SW, YRZ, JHP, and QXO are employees of Nanjing Geneseeq Technology Inc. The remaining authors declare no competing interests.

## References

[1] Petrick J.L., McGlynn K.A. (2019). The changing epidemiology of primary liver cancer. Curr Epidemiol Rep.

[2] Marrero J.A., Kulik L.M., Sirlin C.B. (2018). Diagnosis, staging, and management of hepatocellular carcinoma: 2018 practice guidance by the American Association for the Study of Liver Diseases. Hepatology.

[3] Satyanarayana A., Manns M.P., Rudolph K.L. (2004). Telomeres and telomerase: a dual role in hepatocarcinogenesis. Hepatology.

[4] Schulze K., Nault J.C., Villanueva A. (2016). Genetic profiling of hepatocellular carcinoma using next-generation sequencing. J Hepatol.

[5] Vargas-Rondón N., Villegas V., Rondón-Lagos M. (2017). The role of chromosomal instability in cancer and therapeutic responses. Cancers.

