# Supplementary material for: Comprehensive genomic analysis of early and late-onset hepatocellular carcinoma
Source: Genes Dis. 2023 Jul 12;11(4):101031. doi: 10.1016/j.gendis.2023.06.005 (PMC10950800; doi:10.1016/j.gendis.2023.06.005)
Supplement: Multimedia component 1 [file mmc1.docx]

**Supplementary Materials**

### Methods

### Patients and sample collection

Tumor tissues were collected from primary liver cancer (PLC) patients with a histologic type of HCC admitted to all participating hospitals between October 2016 and May 2020. The age cut-point used in this study that classified early-onset and late-onset HCC was recommended by the 2018 American Association for the Study of Liver Diseases (AASLD) guideline for the management of HCC [[1](#_ENREF_1)]. Specifically, the recommended HCC screening for Asian male and female HBV carriers is 40 and 50 years, respectively. Therefore, we have adopted the same age cut-point in this study to define early-onset and late-onset HCC. Formalin-fixed paraffin-embedded (FFPE) tumors or fresh tumor tissues were confirmed by pathologists at the centralized clinical testing center. Clinical characteristics and treatment history were extracted from medical records. The study was approved by the Medical Ethics Committee of Nanjing Geneseeq Medical Laboratory (NSJB-MEC-2023-02). All participants signed the written informed consent form to use tumor tissue for research purposes. Molecular and clinical data of 159 HCC patients from The Cancer Genome Atlas Program (TCGA) were downloaded from the cBioPortal website (https://www.cbioportal.org/) [[2](#_ENREF_2)]. Overall survival (OS) was defined as the length of time from the date of diagnosis that patients diagnosed with the disease are still alive.

### Genomic DNA extraction

Tumor samples were sent to the central laboratory at Nanjing Geneseeq Technology Inc. (Nanjing, China), a CAP/CLIA (College of American Pathologists and Clinical Laboratory Improvements Amendments) accredited lab, for the following analyses. DNA extraction was performed as previously described [[3](#_ENREF_3), [4](#_ENREF_4)]. FFPE samples were de-paraffinized with xylene followed by genomic DNA extraction using the QIAamp DNA FFPE Tissue Kit (Qiagen Cat. No. 56404) according to the manufacturer’s instructions. Genomic DNA from fresh tumor tissue was extracted using the DNeasy Blood and Tissue Kit (Qiagen Cat. No. 69504) according to standard protocols. Genomic DNA was qualified using Nanodrop2000 (Thermo Fisher Scientific, Waltham, MA), and cfDNA fragment distribution was analyzed on a Bioanalyzer 2100 using the High Sensitivity DNA Kit (Agilent Technologies, Santa Clara, CA, 5067-4626). DNA quantification was performed using the dsDNA HS assay kit on a Qubit 3.0 fluorometer (Life Technology, US). NGS libraries were prepared using the KAPA Hyper Prep kit (KAPA Biosystems) with an optimized manufacturer’s protocol.

### Targeted capture and sequencing

Targeted capture enrichment was performed as previously described [[5](#_ENREF_5)]. In brief, a targeted NGS panel (Nanjing Geneseeq Technology Inc.) covering 425 critical cancer-related genes with a total genomic region of 130 kb was used to identify genomic variants in hotspot exons and regions. According to its internal specifications, the resulting sequencing panel can identify somatic mutations with a sensitivity of 98% and a positive predictive value (PPV) of 95% [[4](#_ENREF_4), [6](#_ENREF_6), [7](#_ENREF_7)]. Library fragment size was determined on Bioanalyzer 2100 (Agilent Technologies). Next-generation sequencing was performed on the Illumina HiSeq 4000 platform (Illumina, San Diego, CA, USA) according to the manufacturer’s instructions.

### Mutation calling

Raw sequencing data were analyzed by a validated automation pipeline. Specifically, raw data were first demultiplexed and subjected to FASTQ file quality control using Trimmomatic [[8](#_ENREF_8)]. Only data without extra nucleotide bases and passed quality control (QC above 15) were retained. Qualified raw reads were mapped to the Human Genome (hg19) using Burrows-Wheeler Aligner (BWA-mem, v0.7.12; <https://github.com/lh3/bwa/tree/master/bwakit>). Local realignment around the insertions/deletions and base quality score recalibration was applied using the Genome Analysis Toolkit (GATK 3.4.0; <https://software.broadinstitute.org/gatk/>). PCR duplicates were removed using Picard. VarScan2 [[9](#_ENREF_9)] was used to identify genetic alterations, including single-nucleotide variations (SNVs) and insertion/deletion mutations. SNVs were filtered out if the variant allele frequency (VAF) was less than 1% for the tumor tissue. Additionally, common SNVs were excluded if they were present in >1% population in the 1000 Genomes Project or the Exome Aggregation Consortium (ExAC) 65,000 exomes database. The resulting mutation list was further filtered by an in-house list of recurrent artifacts based on a normal pool of whole blood samples. Parallel sequencing of matched white blood cells from each patient was performed to remove sequencing artifacts, germline variants, and clonal hematopoiesis. Genomic fusions were identified by FACTERA [[10](#_ENREF_10)] using default parameters (≥2 reads). Fusion reads were manually reviewed and conﬁrmed on the Integrative Genomics Viewer.

Tumor mutation burden (TMB, mutation per Megabase) was determined based on the number of mutations in the targeted regions of the gene panel covering 0.85 Mb of coding genome, excluding known driver mutations as they are over-represented in the panel. Chromosome instability score (CIS) was defined as the proportion of the genome with aberrant (purity-adjusted segment-level copy number ≥3 or ≤1) segmented copy number [[11](#_ENREF_11)]. Somatic mutations were analyzed to identify known cancer driver genes using the OncodriveCLUST method as previously described [[12](#_ENREF_12)]. Co-mutation plot of patients with early- or late-onset HCC showed frequently mutated co-occurring mutations with a mutation frequency ≥5%. For the pathway enrichment analysis, genetic alterations were first categorized into ten signaling pathways associated with cancer hallmarks [[13](#_ENREF_13)]. The proportion of patients harboring mutations in the relevant pathways was compared to reveal the differences in co-existing mutations in patients with early or late-onset HCC.

### Statistical analysis

All statistical analyses were performed using R (version 4.1.3). Fisher's exact test was used to test for associations between categorical variables, and the Wilcoxon-Rank Sum test was used to compare medians of independent groups. Kaplan-Meier curves were used to compare the survival of subgroup patients, and log-rank tests were used to compare the statistical difference. For univariate and multivariate survival analysis, hazard ratios (HR) with 95% confidence intervals (CI) were estimated by the Cox proportional hazards model. A two-sided *P* value of less than 0.05 was considered statistically significant for all tests unless indicated otherwise (**P*<0.05, ***P*<0.01, ****P*<0.001).

## Supplementary Figures


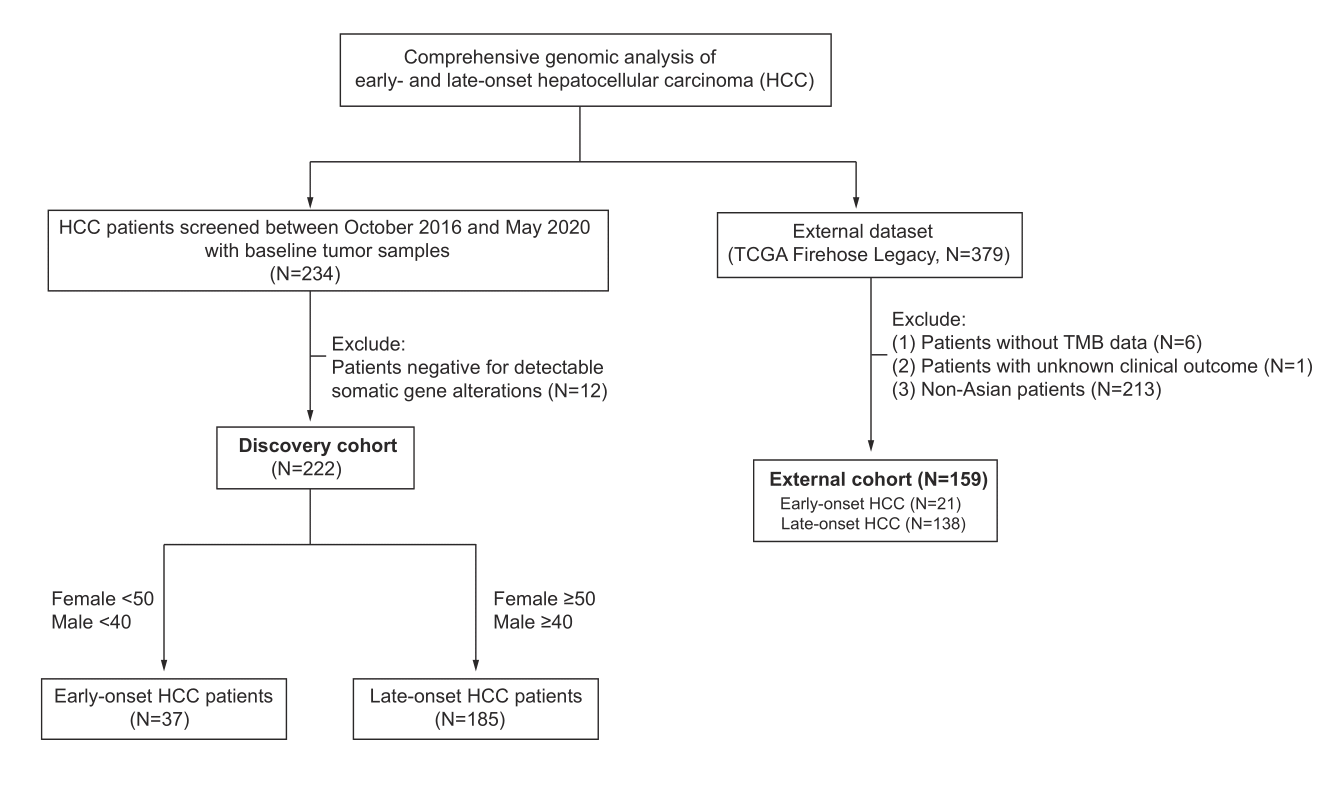


### Figure S1. Patient overview

A total of 234 hepatocellular carcinoma (HCC) patients screened from October 2016 to May 2020 with baseline tumor samples were included in this study. After excluding patients who tested negative for somatic mutations in their baseline tumors, 222 patients were assigned to the discovery cohort, which was then categorized into early-onset and late-onset HCC subgroups based on the American Association for the Study of Liver Diseases (AASLD) guidance (Methods). We have also included an external TCGA dataset comprising mutational and clinical data of 159 Asian HCC patients to investigate the clinical relevance of selected genomic biomarkers in early- and late-onset HCC patients.


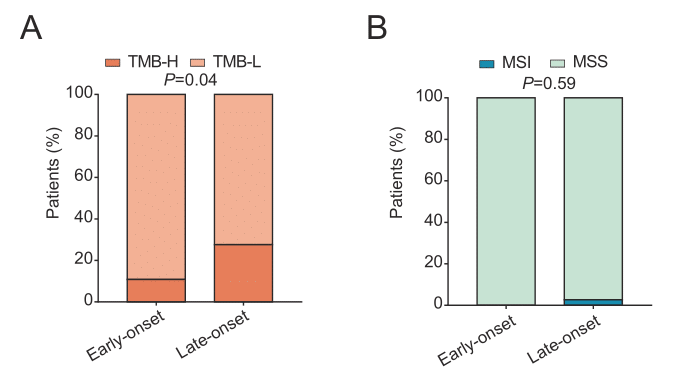


### Figure S2. TMB and MSI analysis of HCC patients.

(A) Bar plot showing the percentage of TMB high (TMB-H) and TMB low (TMB-L) patients within two patient subgroups. (B) Bar plot demonstrating the proportion of microsatellite instability (MSI) and microsatellite stable (MSS) patients within two patient subgroups.


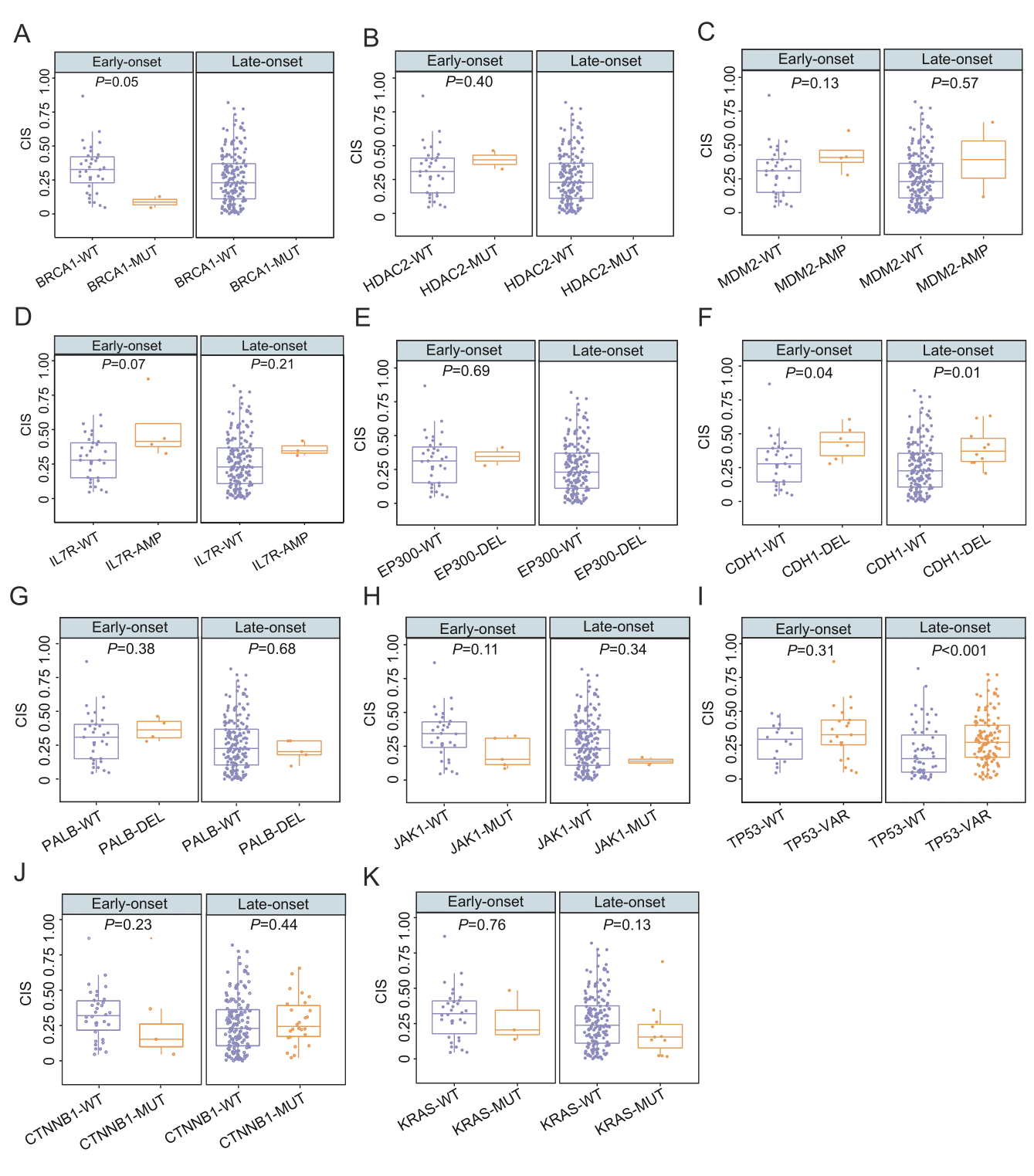


### Figure S3. CIS of patients within two patient subgroups.

Box plots illustrating the chromosomal instability score (CIS) of patients with early-onset or late-onset HCC harboring specified genetic alterations.


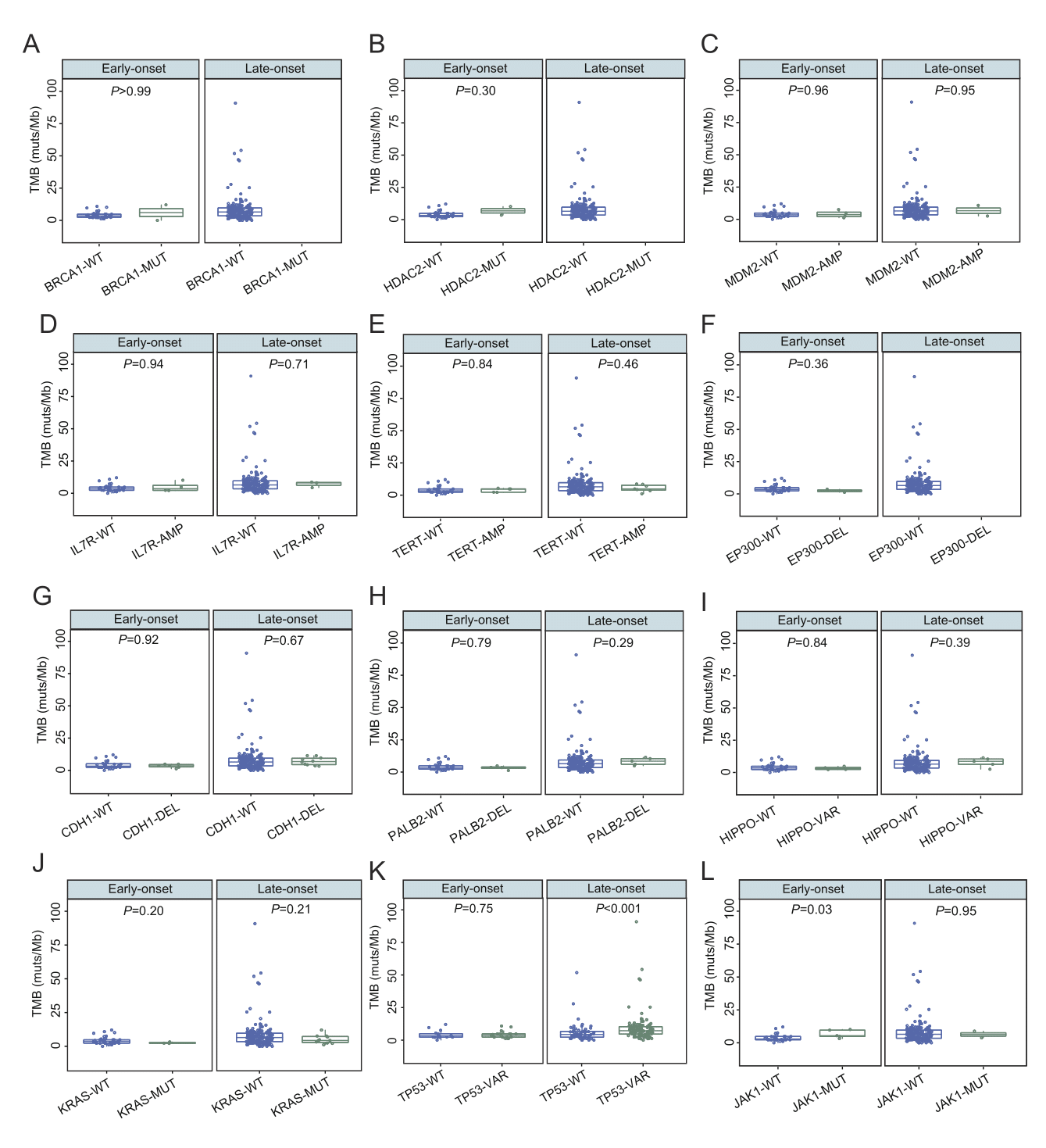


### Figure S4. TMB of patients within two patient subgroups.

Box plots illustrating the tumor mutation burden (TMB) of patients with early-onset or late-onset HCC harboring specified genetic alterations.


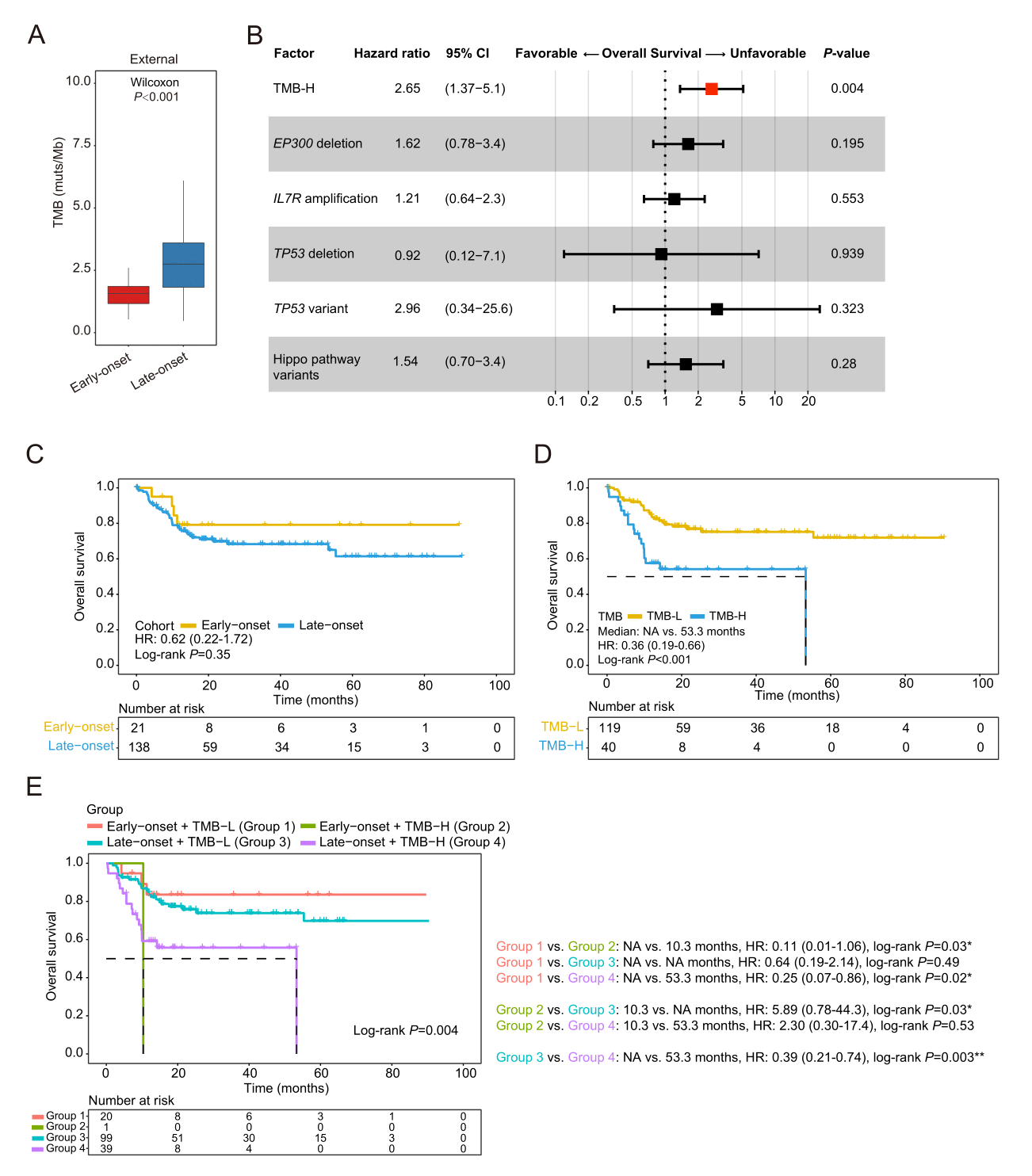


### Figure S5. Survival analyses using the external TCGA cohort.

(A) Distribution of tumor mutation burden (TMB) in early-onset (N=21) and late-onset patients (N=138) with HCC. (B) Multivariate survival analysis of genetic biomarkers and their association with the overall survival of HCC patients. (C-D) Kaplan-Meier plot showing the overall survival of HCC patients stratified by disease onset age (C) and TMB status (D). (E) Kaplan-Meier plot demonstrating the overall survival of early- or late-onset HCC patients stratified by TMB status.

## Supplementary Tables

| **Table S1. Clinical characteristics of this study cohort (N=222)** | | | | |
| --- | --- | --- | --- | --- |
| Characteristics | Total  (N=222) | Early-onset  (N=37) | Late-onset (N=185) | *P*-value |
| Age at diagnosis (years) |  |  |  | <0.001^a^ |
| Median | 55 | 36 | 57 |  |
| Range | 19-80 | 26-49 | 40-78 |  |
| Sex |  |  |  | <0.001^b^ |
| Female | 37 (16.7%) | 16 (43.2%) | 21 (11.4%) |  |
| Male | 185 (83.3%) | 21 (56.8%) | 164 (88.6%) |  |
| Risk factor |  |  |  | >0.99^b^ |
| Hepatitis B | 47 (21.2%) | 8 (21.6%) | 39 (21.1%) |  |
| Unknown | 175 (78.8%) | 29 (78.4%) | 146 (78.9%) |  |
| Clinical stage |  |  |  | 0.48^b^ |
| I-III | 25 (11.3%) | 6 (16.2%) | 19 (10.3%) |  |
| Ⅳ | 30 (13.5%) | 4 (10.8%) | 26 (14.0%) |  |
| Unknown | 167 (75.2%) | 27 (73.0%) | 140 (75.7%) |  |
| Family history of cancer |  |  |  | >0.99^b^ |
| No | 157 (70.7%) | 26 (70.3%) | 131 (70.8%) |  |
| Yes | 65 (29.3%) | 11 (29.7%) | 54 (29.2%) |  |
| Family history of primary liver cancer | |  |  | 0.47^b^ |
| No | 185 (83.3%) | 29 (78.4%) | 156 (84.3%) |  |
| Yes | 37 (16.7%) | 8 (21.6%) | 29 (15.7%) |  |
| ^a^The median diagnosis age of patients was calculated by the Wilcoxon-Rank Sum test.  ^b^Fisher’s exact test was used to compare the distribution of categorical variables between early- and late-onset hepatocellular carcinoma patients. | | | | |

| **Table S2. Clinical characteristics of patients in the external TCGA dataset (N=159)** | | | | |
| --- | --- | --- | --- | --- |
| Characteristic | Total  (N=159) | Early-onset (N=21) | Late-onset (N=138) | *P*-value |
| Age at diagnosis (years) |  |  |  | <0.001^a^ |
| Median | 55 | 38 | 58 |  |
| Range | 18-76 | 18-49 | 40-76 |  |
| Sex |  |  |  | 0.08^b^ |
| Female | 34 (21.4%) | 8 (38.1%) | 26 (18.8%) |  |
| Male | 125 (78.6%) | 13 (61.9%) | 112 (81.2%) |  |
| Histology |  |  |  | >0.99^b^ |
| HCC | 156 (98.1%) | 21 (100%) | 135 (97.8%) |  |
| HCC + ICC | 3 (1.9%) | 0 (0.00%) | 3 (2.17%) |  |
| Clinical stage |  |  |  |  |
| I | 81 (50.9%) | 9 (42.9%) | 72 (52.2%) | 0.67^b^ |
| II | 34 (21.4%) | 4 (19.0%) | 30 (21.7%) |  |
| III | 41 (25.8%) | 8 (38.1%) | 33 (23.9%) |  |
| IV | 1 (0.63%) | 0 (0.00%) | 1 (0.72%) |  |
| Unknown | 2 (1.26%) | 0 (0.00%) | 2 (1.45%) |  |
| Family history of cancer |  |  |  | >0.99^b^ |
| No | 127 (79.9%) | 15 (71.4%) | 112 (81.2%) |  |
| Yes | 19 (11.9%) | 2 (9.52%) | 17 (12.3%) |  |
| Unknown | 13 (8.2%) | 4 (19.0%) | 9 (6.52%) |  |
| Risk factor |  |  |  | 0.22^b^ |
| Alcohol consumption | 25 (15.7%) | 2 (9.52%) | 23 (16.7%) |  |
| Alcohol consumption/HBV | 18 (11.3%) | 0 (0.00%) | 18 (13.0%) |  |
| Alcohol consumption/HCV | 1 (0.6%) | 0 (0.00%) | 1 (0.72%) |  |
| HBV | 70 (44.0%) | 11 (52.4%) | 59 (42.8%) |  |
| HCV | 5 (3.1%) | 0 (0.00%) | 5 (3.62%) |  |
| HBV/HCV | 2 (1.3%) | 0 (0.00%) | 2 (1.45%) |  |
| Non-alcoholic fatty liver disease | 1 (0.6%) | 0 (0.00%) | 1 (0.72%) |  |
| No History | 25 (15.7%) | 7 (33.3%) | 18 (13.0%) |  |
| Unknown | 12 (7.6%) | 1 (4.76%) | 11 (7.97%) |  |
| ^a^The median diagnosis age of patients was calculated by the Wilcoxon-Rank Sum test.  ^b^*P*-values were based on Fisher’s exact test to compare the distribution of categorical variables between early- and late-onset hepatocellular carcinoma patients.  Abbreviations: HCC, hepatocellular carcinoma; ICC, intrahepatic cholangiocarcinoma; HBV, hepatitis B virus; HCV, hepatitis C virus. | | | | |

| **Table S3. Comparing the clinical context between our study cohort and the external dataset** | | | |
| --- | --- | --- | --- |
| Characteristic | This study (N=222) | External (N=159) | *P*-value |
| Age at diagnosis (years) |  |  | 0.42^a^ |
| Median | 55 | 55 |  |
| Range | 19-80 | 18-76 |  |
| Sex |  |  | 0.29^b^ |
| Female | 37 (16.7%) | 34 (21.4%) |  |
| Male | 185 (83.3%) | 125 (78.6%) |  |
| Disease onset |  |  | 0.39^b^ |
| Early-onset | 37 (16.7%) | 21 (13.2%) |  |
| Late-onset | 185 (83.3%) | 138 (86.8%) |  |
| Clinical stage |  |  | NA |
| I-III | 25 (11.3%) | 156 (98.1%) |  |
| IV | 30 (13.5%) | 1 (0.63%) |  |
| Unknown | 167 (75.2%) | 2 (1.26%) |  |
| Family history of cancer |  |  | <0.001^b^ |
| No | 157 (70.7%) | 127 (79.9%) |  |
| Yes | 65 (29.3%) | 19 (11.9%) |  |
| Unknown | 0 (0.00%) | 13 (8.2%) |  |
| ^a^The median diagnosis age of patients was calculated by the Wilcoxon-Rank Sum test.  ^b^Fisher’s exact test was used to compare the distribution of categorical variables between the two datasets. | | | |

| **Table S4. Univariate survival analysis of factors of overall survival in the TCGA dataset** | | |
| --- | --- | --- |
| Biomarkers | HR (95% CI) | *P*-value^a^ |
| TMB (TMB-H vs. TMB-L) | 2.8 (1.5-5.2) | **<0.001** |
| Sex (male vs. female) | 0.87 (0.42-1.8) | 0.71 |
| Age at diagnosis (EO vs. LO) | 1.6 (0.58-4.5) | 0.35 |
| Family history of cancer (yes vs. no) | 0.58 (0.21-1.65) | 0.31 |
| *JAK1* mutation | 1.3 (0.42-4.3) | 0.62 |
| *CDH1* deletion | 1.8 (0.94-3.3) | 0.08 |
| *EP300* deletion | 2.8 (1.4-5.5) | **0.003** |
| *IL7R* amplification | 1.9 (1-3.4) | **0.04** |
| *MDM2* amplification | 1.8 (0.82-3.8) | 0.15 |
| *PALB2* deletion | 1.4 (0.78-2.7) | 0.25 |
| *TERT* amplification | 1.8 (0.99-3.3) | 0.06 |
| *CTNNB1* mutation | 0.94 (0.47-1.9) | 0.85 |
| *KRAS* mutation | 2.2 (0.3-16) | 0.45 |
| *TP53* mutation | 1.6 (0.86-2.8) | 0.14 |
| *TP53* deletion | 2.6 (1.2-5.5) | **0.02** |
| *TP53* variant | 2.9 (1.3-6.5) | **0.01** |
| Hippo pathway variants | 2.5 (1.2-5.1) | **0.02** |
| ^a^Bold represents significant *P*-values calculated based on the Cox proportional hazards model.  Abbreviations: HR, hazard ratio; CI, confidence interval; EO, early-onset; LO, late-onset | | |

| **Table S5. Potentially actionable drug targets in HCC patients.** | | |
| --- | --- | --- |
| Targets | Drugs | Source |
| ATM | Olaparib | OncoKB |
| BRAF | Vemurafenib + Atezolizumab + Cobimetinib | OncoKB |
| BRCA2 | Niraparib | OncoKB |
| CDK12 | Olaparib | OncoKB |
| EGFR | Afatinib | OncoKB |
| ERBB2 | Ado-Trastuzumab Emtansine | OncoKB |
| FGFR2 | Erdafitinib | OncoKB |
| IDH1 | Ivosidenib | OncoKB |
| KRAS | Sotorasib | OncoKB |
| MET | Capmatinib | OncoKB |
| NF1 | Selumetinib | OncoKB |
| NRAS | Panitumumab, Panitumumab + Chemotherapy | OncoKB |
| NTRK1 | Entrectinib | OncoKB |
| PDGFRA | Avapritinib | OncoKB |
| PIK3CA | Alpelisib + Fulvestrant | OncoKB |
| TSC1 | Everolimus | OncoKB |
| TSC2 | Everolimus | OncoKB |
| AKT1 | AZD5363 | OncoKB |
| ERCC2 | Cisplatin | OncoKB |
| ESR1 | AZD9496, Fulvestrant | OncoKB |
| FGFR1 | Debio1347, Infigratinib, Erdafitinib | OncoKB |
| HRAS | Tipifarnib | OncoKB |
| MAP2K1 | Trametinib, Cobimetinib | OncoKB |
| MTOR | Everolimus | OncoKB |
| ARID1A | PLX2853 | OncoKB |
| CDK4 | Palbociclib, Abemaciclib | OncoKB |
| CDKN2A | Palbociclib, Ribociclib, Abemaciclib | OncoKB |
| MDM2 | Milademetan | OncoKB |
| PTEN | GSK2636771, AZD8186 | OncoKB |
| STK11 | Bemcentinib + Pembrolizumab | OncoKB |
| FGF19 | fisogatinib | BLU-554-1101 |
| FGFR4 | fisogatinib | BLU-554-1101 |

## References

1. J.A. Marrero, L.M. Kulik, C.B. Sirlin, A.X. Zhu, R.S. Finn, M.M. Abecassis, et al., *Diagnosis, Staging, and Management of Hepatocellular Carcinoma: 2018 Practice Guidance by the American Association for the Study of Liver Diseases.* Hepatology, 2018. **68**(2): p. 723-750.

2. E. Cerami, J. Gao, U. Dogrusoz, B.E. Gross, S.O. Sumer, B.A. Aksoy, et al., *The cBio cancer genomics portal: an open platform for exploring multidimensional cancer genomics data.* Cancer Discov, 2012. **2**(5): p. 401-4.

3. Z. Yang, N. Yang, Q. Ou, Y. Xiang, T. Jiang, X. Wu, et al., *Investigating Novel Resistance Mechanisms to Third-Generation EGFR Tyrosine Kinase Inhibitor Osimertinib in Non-Small Cell Lung Cancer Patients.* Clin Cancer Res, 2018. **24**(13): p. 3097-3107.

4. Y. Shu, X. Wu, X. Tong, X. Wang, Z. Chang, Y. Mao, et al., *Circulating Tumor DNA Mutation Profiling by Targeted Next Generation Sequencing Provides Guidance for Personalized Treatments in Multiple Cancer Types.* Sci Rep, 2017. **7**(1): p. 583.

5. W. Fang, Y. Ma, J.C. Yin, S. Hong, H. Zhou, A. Wang, et al., *Comprehensive Genomic Profiling Identifies Novel Genetic Predictors of Response to Anti-PD-(L)1 Therapies in Non-Small Cell Lung Cancer.* Clin Cancer Res, 2019. **25**(16): p. 5015-5026.

6. K. Clifton, T.A. Rich, C. Parseghian, V.M. Raymond, A. Dasari, A.A.L. Pereira, et al., *Identification of Actionable Fusions as an Anti-EGFR Resistance Mechanism Using a Circulating Tumor DNA Assay.* JCO Precis Oncol, 2019. **3**.

7. Y. Jin, H. Bao, X. Le, X. Fan, M. Tang, X. Shi, et al., *Distinct co-acquired alterations and genomic evolution during TKI treatment in non-small-cell lung cancer patients with or without acquired T790M mutation.* Oncogene, 2020. **39**(9): p. 1846-1859.

8. A.M. Bolger, M. Lohse and B. Usadel, *Trimmomatic: a flexible trimmer for Illumina sequence data.* Bioinformatics, 2014. **30**(15): p. 2114-20.

9. E. Reble, C.A. Castellani, M.G. Melka, R. O'Reilly and S.M. Singh, *VarScan2 analysis of de novo variants in monozygotic twins discordant for schizophrenia.* Psychiatr Genet, 2017. **27**(2): p. 62-70.

10. A.M. Newman, S.V. Bratman, H. Stehr, L.J. Lee, C.L. Liu, M. Diehn and A.A. Alizadeh, *FACTERA: a practical method for the discovery of genomic rearrangements at breakpoint resolution.* Bioinformatics, 2014. **30**(23): p. 3390-3.

11. S. Turajlic, H. Xu, K. Litchfield, A. Rowan, T. Chambers, J.I. Lopez, et al., *Tracking Cancer Evolution Reveals Constrained Routes to Metastases: TRACERx Renal.* Cell, 2018. **173**(3): p. 581-594 e12.

12. D. Tamborero, A. Gonzalez-Perez and N. Lopez-Bigas, *OncodriveCLUST: exploiting the positional clustering of somatic mutations to identify cancer genes.* Bioinformatics, 2013. **29**(18): p. 2238-44.

13. F. Sanchez-Vega, M. Mina, J. Armenia, W.K. Chatila, A. Luna, K.C. La, et al., *Oncogenic Signaling Pathways in The Cancer Genome Atlas.* Cell, 2018. **173**(2): p. 321-337 e10.
